# Patterns of intestinal parasite prevalence in brown bears (*Ursus arctos*) revealed by a 3-year survey on the Shiretoko peninsula, Hokkaido, Japan

**DOI:** 10.1016/j.ijppaw.2025.101048

**Published:** 2025-02-19

**Authors:** Mizuki Moriyoshi, Naoki Hayashi, Nariaki Nonaka, Ryo Nakao, Masami Yamanaka, Toshio Tsubota, Michito Shimozuru

**Affiliations:** a Laboratory of Wildlife Biology and Medicine, Faculty of Veterinary Medicine, Hokkaido University, Kita-18, Nishi-9, Kita-ku, Sapporo, Hokkaido, 060-0818, Japan; b Laboratory of Parasitology, Faculty of Veterinary Medicine, Hokkaido University, Kita-18, Nishi-9, Kita-ku, Sapporo, Hokkaido, 060-0818, Japan; c Division of Parasitology, Veterinary Research Unit, International Institute for Zoonosis Control, Hokkaido University, Kita-18, Nishi-9, Kita-ku, Sapporo, Hokkaido, 060-0818, Japan; d One Health Research Center, Hokkaido University, Kita-18, Nishi-9, Kita-ku, Sapporo, Hokkaido, 060-0818, Japan; e Shiretoko Nature Foundation, 531 Iwaubetsu, Shari, Hokkaido, 099-4356, Japan

**Keywords:** *Ursus arctos*, Intestinal parasite, *Dibothriocephalus nihonkaiensis*, *Baylisascaris transfuga*, *Uncinaria* sp., Seasonality

## Abstract

This study examined the parasite fauna of wild brown bears and differences in the likelihood of parasite detection by season (summer vs autumn), year, and host factors (sex and age class). From June 2022 to November 2024, 334 fecal samples were collected from the Shiretoko Peninsula, Hokkaido, Japan, and examined for parasites using the centrifugal flotation technique. Fecal DNA analysis and data from a long-term field monitoring survey led to the identification of 49 individuals, which were further classified based on sex and age. Parasites detected in the feces included *Uncinaria* sp. (35.0% of total samples), *Baylisascaris transfuga* (13.5%), *Dibothriocephalus nihonkaiensis* (8.4%), Strongylida (2.7%), Capillariidae (0.6%), and coccidia (0.6%). The prevalence of detection for *D. nihonkaiensis* tended to be higher in autumn (September–November) than in summer (May–August). The likelihood of *B. transfuga* and *Uncinaria* sp. detection varied by season, year, and bear age class. The likelihood of *B. transfuga* detection was significantly higher in autumn than in summer, and tended to be higher in young bears (0–2 years) than in subadult/adult (≥3 years) bears, whereas that of *Uncinaria* sp. was significantly higher in summer than in autumn and in subadult/adult than in young bears. Egg shedding by these three parasites tended to disappear before or during hibernation. These results suggest that the likelihood of parasite detection in brown bear reflects the interactions of environmental and host factors, including seasonal and/or annual changes in diet, winter hibernation, and host growth.

## Highlights

• *Uncinaria* sp., *B. transfuga*, and *D. nihonkaiensis* are common in brown bears in Japan.
• Parasite presence varied by seasons (summer/autumn), years and age class of bears.
• Egg shedding of endoparasites tended to disappear before or during hibernation.
• Seasonal and annual variations in prevalence are related to their feeding ecology.

## Graphical abstract

**Figure:**
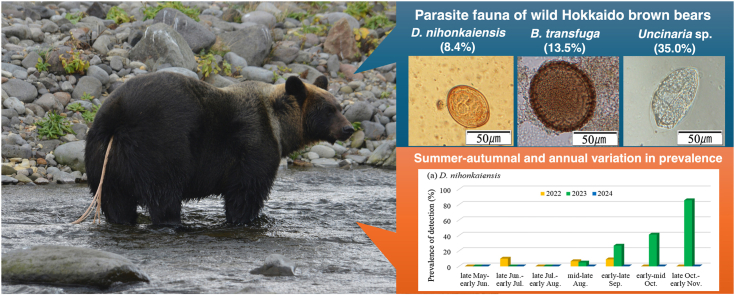

## 1 Introduction

Parasite infection may be harmful to the health of wildlife and threaten both endangered species and small populations. Poor nutritional status of the host induced by parasite infection can result in a reduced reproductive efficiency and negative effects on population growth (Stien et al., 2002). Implementing the appropriate wildlife conservation and management measures requires knowledge of the infection patterns and ecology of parasites. This is especially crucial for parasites in wildlife that can also infect humans, livestock, and companion animals. The risk of zoonoses, including parasitic diseases, is expected to increase as urbanization increases the proximity between humans and wildlife (Graeff-Teixeira et al., 2016; 7Hassell et al., 2017). At the same time, parasites are integral components of biological communities and play an important role in ecosystem functioning (Thompson et al., 2010). Collecting data on parasite species in wildlife hosts and understanding their life cycles, biology, infection routes, and prevalence is crucial. This information is vital not only for public health but also for biodiversity conservation. However, such data remain lacking for many parasites of wildlife.

Brown bears (*Ursus arctos*) are distributed throughout Eurasia and North America. In Japan, the Hokkaido brown bear (*U. a. yesoensis*) subspecies inhabits only Hokkaido, the northernmost island of Japan. The Hokkaido brown bear population has increased over the past 30 years (Hokkaido Government, Japan, 2022), such that the bears now have home ranges near urban areas (i.e., “urban bears”), which has caused serious conflicts with people (Sato, 2017). The brown bear is the largest terrestrial mammal in Japan, with a home range that spans from tens to hundreds of square kilometers (Dahle and Swenson, 2003; Støen et al., 2005; Sato et al., 2008), and thus has the potential to carry numerous bacterial and parasitic pathogens over a broad area. Indeed, bears play an important role as reservoirs and vectors for several parasitic diseases (Di Salvo and Chomel, 2020). The brown bear is an omnivore that feeds on various food items depending on the season, including grasses, berries, insects, and deer from spring to early summer, stone pine cones and salmon from summer to autumn, and acorns and fruits in late autumn (Sato et al., 2005a, Sato et al., 2005b; Shirane et al., 2021); thus, the diverse feeding habits of the brown bear may enhance its risk of parasite infection.

Several studies have investigated the intestinal parasites of brown bears in Eurasia and North America. Cano et al. (2024) recently reported variations in parasites of Cantabrian brown bears based on 248 fecal samples collected year-round, covering all four seasons. By contrast, only a few reports based on small sample sizes, collected over limited periods, are available for Hokkaido, Japan. To date, the parasites *Dibothriocephalus nihonkaiensis* (Sasaki et al., 2019; Ohta et al., 2021), *Ancylostoma malayanum* (Asakawa et al., 2006), *Uncinaria* sp. (Asakawa, 2024), and *Baylisascaris transfuga* (Inukai and Yamashita, 1934; Asakawa, 2024) have been reported in wild brown bears in Hokkaido, whereas brown bear parasites in Eurasia and North America include nematodes such as *B. transfuga*, *Uncinaria* species, Strongylida, and Capillariidae; cestodes such as Diphyllobothriidae and *Taenia* species; trematodes such as *Dicrocoelium* species; and coccidia (Gau et al., 1999; Foster et al., 2004; Borka-Vitális et al., 2017; Bugmyrin et al., 2017). Importantly, some of these parasites can infect humans and domestic animals; for example, *D. nihonkaiensis* is well known to infect humans (Arizono et al., 2009). *Baylisascaris transfuga*, a common parasite of brown bears (Bauer, 2013; Catalano et al., 2015a; Remesar et al., 2024), is potentially zoonotic (Samuel et al., 2001), although it is less pathogenic than *B. procyonis*, which cause serious larva migrans in humans and many wild mammals (Sapp et al., 2017).

The life cycles of brown bear parasites are also largely unknown. Previous studies have shown that intestinal parasites in brown bears are most common in autumn and less common in spring, which suggests that bears lose their intestinal parasites during or before hibernation (Gau et al., 1999; Remesar et al., 2024). However, little is known about the seasonal and yearly differences in parasite infection and the factors responsible for such differences (e.g., feeding items). In addition, most studies have been based on carcasses or fecal samples, providing only “snapshot” information about the moment when the samples were obtained. This makes it difficult to elucidate infection status dynamics at the individual level and to identify host factors (e.g., sex and age) that influence parasite load of individuals. In this study, we aimed to identify the parasite fauna of wild Hokkaido brown bears and to evaluate differences in the likelihood of parasite detection by season (summer vs autumn), year, and host factors (sex and age class). To accomplish this, the Rusha area of the Shiretoko Peninsula, Hokkaido, Japan, was selected as the study site where a long-term bear monitoring survey has been conducted since the late 1990s and sex-age information of identified bears is available (Kohira et al., 2009; Shimozuru et al., 2017; Shirane et al., 2021).

## 2 Materials and methods

### 2.1 Study area

**Fig. 1:**
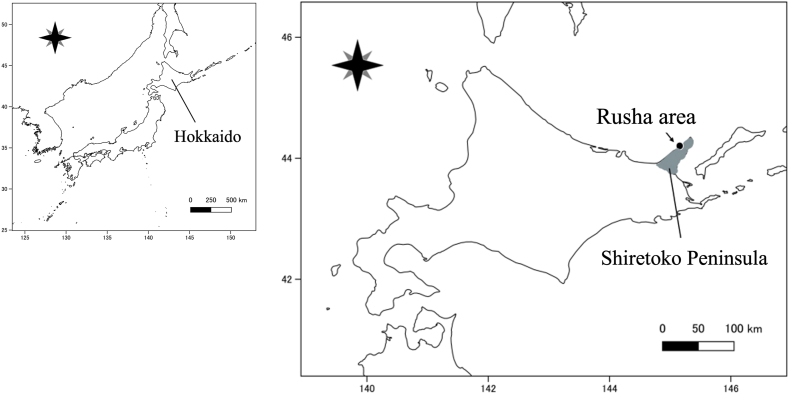
Map of the study area in Hokkaido, Japan, including the Rusha area (black dot) of the Shiretoko Peninsula (shaded).

The Shiretoko Peninsula, located in eastern Hokkaido, Japan, has one of the highest densities of brown bears in the world, with 559 ± 440 bears (mean ± 95% confidence interval [CI]) within 1760 km^2^ (i.e. 32 bears/100 km^2^; Ministry of the EnvironmentGovernment of Japan, 2017). An area of 610 km^2^, from the center to the tip of the Peninsula, has been designated as a national park, and an area of 711 km^2^ that includes the national park together with the surrounding terrestrial and marine areas was registered as a UNESCO World Natural Heritage Site in 2005. The Rusha area (44°11′N, 145°11′E; approximately 11.5 km^2^), where the study was conducted, is located near the tip of the peninsula (Fig. 1) and is a special wildlife protection area; public access is not allowed without permission. Rusha includes an estuarine coast extending approximately 3 km from north to south. At sites where rivers flow into the sea, pink salmon (*Oncorhynchus gorbuscha*) and chum salmon (*Oncorhynchus keta*) spawn beginning in August (Nakamura and Komiyama, 2010). Long-term brown bear monitoring surveys have been conducted in the Rusha area since 1997, and data from previous studies on the diet and population structure of the bears in this region are available (Shimozuru et al., 2017; Shirane et al., 2021). Furthermore, many individual bears are identifiable visually and/or using DNA-based methods that provide information on the age, sex, and parentage relationships of the bears (Shimozuru et al., 2019, 2022). Thus, in addition to the parasitic infection status, information on individual hosts can be extracted by fecal analyses. The high bear density of the Rusha area, with an average of 40 individuals per year, including about 15 philopatric females and their offspring, was confirmed in a previous study (Shimozuru et al., 2017). This high density allows for efficient fecal collection. In addition, the presence of philopatric females has enabled continuous, long-term research at the individual level.

### 2.2 Fecal sample collection

Bear feces were collected from June 2022 to November 2024, mainly in grasslands and coasts in the Rusha area but also along survey roads leading to the area (approximately 9.5 km). For each fecal sample, the following information was recorded: collection date and location, estimated days since defecation, and percent volume of each food item estimated visually, as described in a previous study (Shirane et al., 2021). For DNA-based individual identification and parasitological examination, feces with ages of 0–5 days as estimated by field collectors were collected. For feces used in DNA-based individual identification, after the surface had been scrubbed with a flocked swab (Flocked Swab R30; Sugiyama-gen Co., Ltd., Tokyo, Japan), the sample was stored in 2.0-mL tubes with Inhibitex buffer (Qiagen Inc., Tokyo, Japan) at −30 °C until DNA extraction (Shimozuru et al., 2022). For parasitological examination, the same fecal samples were placed in 50-mL centrifuge tubes and stored at 4 °C until analysis. To reduce the risk of soil contamination, the samples were collected from inside the fecal mass using disposable chopsticks. Multiple pieces of feces found in close proximity were considered to be derived from the same individual if they were similar in content and freshness. Fecal samples were collected primarily by one skilled staff member, although sometimes by two individuals who had received thorough instruction from him. If the period between sample collection and parasitological examination was expected to exceed 2 weeks, the samples were stored at −30 °C until analysis. Some fecal samples were collected directly from the rectum of captured bears at the time of their capture (Nakamura et al., 2023) and used for research.

### 2.3 Diet estimation

The percent volume of each food item in the fecal sample was visually estimated and recorded for each sampling period, as described in a previous study (Shirane et al., 2021). Because different foods have different digestibility rates, the corresponding correction factors (CF; Hewitt and Robbins, 1996; Dahle et al., 1998; Persson et al., 2001; Bojarska and Selva, 2012; Stenset et al., 2016) were applied, as also described previously (Shirane et al., 2021). Eight categories were defined: plants (grass and leaves), pine nuts (cones of Japanese stone pine, *Pinus pumila*), drupes (e.g., *Prunus ssiori*), berries (e.g., wild vine, *Vitis coignetiae* and hardy kiwi, *Actinidia* spp.), acorns and other nuts (e.g., Japanese oak, *Quercus crispula,* and Manchurian walnut, *Juglans mandshurica*), ants, salmon (pink salmon and chum salmon), and others (e.g., sika deer, *Cervus nippon yesoensis*).

### 2.4 DNA-based individual identification

DNA-based individual identification was conducted according to Shimozuru et al. (2022). Briefly, fecal DNA was extracted using the QIAamp Fast DNA stool mini kit (Qiagen Inc.). Each DNA sample was tested in a multiplex polymerase chain reaction (PCR) assay using two primer mixes including six microsatellite markers (Primer A; G1A, MU05, and MU51, Primer B; MU50, G10B, and MU23). Allele size was determined using the SeqStudio genetic analyzer (Thermo Fisher Scientific K.K., Tokyo, Japan). Individual bears were genetically identified and their age and sex were determined by comparing the results of microsatellite analysis with the database obtained during long-term monitoring between the late 1990s and 2024 (Kohira et al., 2009; Shimozuru et al., 2017, 2022). When a sample did not match precisely with any individuals recorded to date, it was tested with 21 microsatellite markers, including the above six markers, and one sex marker, amelogenin (Yamamoto et al., 2002). Parentage relationships were determined using a likelihood-based approach with the CERVUS software (version 3.0.7) (Kalinowski et al., 2007). The reproductive history of adult females has been monitored as part of the field study, allowing the age of their offspring to be identified. If age could not be determined, then the age class (i.e., cub from that year, 1 year old, or older) was estimated based on body size (if available) and breeding experience (i.e., previous offspring documented in the data set) (Shimozuru et al., 2022).

### 2.5 Parasitological examinations

Parasite eggs or oocysts were detected using the centrifugal flotation technique (Ito, 1980) for all fecal samples. Briefly, feces were weighed and 2–5 g from each sample was mixed with 20 mL of water and strained through a metal mesh with a pore size of 150 μm. The filtrate was transferred to two 15-mL centrifuge tubes and centrifuged at 1050×*g* for 5 min. The supernatant was discarded and the sediment was mixed with a sucrose solution (specific gravity: 1.27) and centrifuged at 1050×*g* for 10 min. Sucrose solution was again added until the tubes were filled and a cover-slip was placed on the tube. After 10 min, the cover-slip was examined under a microscope. The Mini-FLOTAC technique (Cringoli et al., 2013) with sucrose solution (specific gravity: 1.27) was applied to count and quantify eggs or oocysts for samples in which parasites were detected. Based on the number of eggs or oocysts detected by the centrifugal flotation technique, 0.5–2.5 g of feces were weighed, and then dissolved in 10 ml of sucrose solution, according to the same procedure as described above. Samples with particularly large numbers of eggs were further diluted for quantification of eggs. Two milliliters of the solution were extracted, with 1 mL used to fill each of the two chambers of the Mini-FLOTAC. After a 10-min settling period, the eggs or oocysts in each chamber were counted. The number of eggs per gram (EPG) or oocysts per gram (OPG) for each sample were calculated based on the average value of the counts, total volume of the solution, and weight of the feces analyzed.

Prevalence of detection and prevalence of infection were determined for each parasite and each period. Prevalence of detection were calculated by dividing the number of feces in which eggs or oocysts were detected by the total number of feces. The prevalence of infection was defined as the number of infected bears divided by the total number of bears identified by microsatellite analysis.

### 2.6 Parasite identification

Molecular identification was performed on frequently detected eggs, including hookworm, roundworm, and Diphyllobothriidae. Samples were selected to ensure that there were no duplicates from the same individual in the same year. Eggs were isolated from feces using the flotation method. The egg-containing supernatant (2 mL) was transferred into a centrifuge tube, mixed with 12 mL of water, and centrifuged at 1050×*g* for 5 min. The supernatant was discarded; the sediment was dissolved in approximately 1 mL of remaining water, transferred to a 1.5-mL tube and centrifuged at 1050×*g* for 10 min. The resulting supernatant was discarded, and the remainder of the sample (in approximately 50 μL of water), containing eggs, was stored at −30 °C until DNA extraction.

Egg samples were frozen at −80 °C for 5 min and thawed at 37 °C for 1 min, which was repeated for five cycles. The sample was then transferred to a tube with 0.1 mm glass beads, crushed at 3000 rpm for 1 min, and centrifuged at 21,500×*g* for 1 min. Twenty μL of supernatant was transferred to a 1.5-mL tube and DNA was extracted using the phenol-chloroform method (Irie et al., 2017). The DNA was purified using NucleoSpin gel and the PCR Clean-up kit (MACHEREY-NAGEL, Germany), eluted in 20 μL of TE buffer, and stored at −30 °C.

For hookworms, the internal transcribed spacer 1 (ITS-1) region of nuclear ribosomal DNA (rDNA) was amplified using primers 93 and 264 (Nadler et al., 2000a). For roundworms and Diphyllobothriidae, the cytochrome *c* oxidase subunit 1 (*cox1*) gene of mitochondrial DNA (mtDNA) was amplified using the BpCox1-F1 and BpCox1-R1 primers (Sato et al., 2005a, Sato et al., 2005b) and the BW3 and BW4.5 primers (Wicht et al., 2007), respectively. Amplification was performed in a 25.0 μL of reaction mixture containing 12.5 μL of 2 × Gflex PCR buffer (TaKaRa Bio Inc., Kusatsu, Japan), 1 mM of Mg^2+^, 200 μM of each dNTP, 0.2 μM of each primer, 0.625 unit of Tks Gflex DNA Polymerase (TaKaRa Bio Inc.), 1.0 μL of DNA template, and 10.0 μL of DDW. After 1 min at 94 °C, the amplification was performed for 40 cycles of 10 s at 98 °C, 15 s at 56 °C for hookworms, 52 °C for roundworms, or 55 °C for Diphyllobothriidae, and 30 s at 68 °C, with a final extension step of 2 min at 68 °C. Agarose gel electrophoresis was performed to confirm that the target genes were amplified. The PCR products were purified using FastGene™ Gel/PCR Extraction kit (NIPPON Genetics Co., Ltd., Tokyo, Japan) and then were sequenced using BigDye™ Terminator v3.1 Cycle Sequencing Kit (Thermo Fisher Scientific K. K.) with SeqStudio Genetic Analyzer and BioEdit version 7.7.1. The same primers were used for sequencing. The obtained sequences were compared with the registered sequences in the National Center for Biotechnology Information (NCBI) GenBank database. A phylogenetic analysis of *Uncinaria* species was performed using MrBayes v 3.2.6 (Ronquist et al., 2012). in MrBayes5D (Tanabe, 2008) under K80 model with 1,000,000 generations. The analysis included the ITS-1 sequences of our *Uncinaria* samples and other hookworms deposited in GenBank (AF194145, HQ262052, KJ026500, KJ026495, HQ262132, HQ262120, and JQ812694; Nadler et al., 2000b, 2013; Lucio-Forster et al., 2012; Catalano et al., 2015b). All sequences were aligned by MAFFT v7.450 software (Katoh and Standley, 2013) and then trimmed to match the shortest sequence. The ITS-1 sequence of *Ancylostoma caninum* (JQ812694) was used as an outgroup. The consensus tree was visualized by FigTree v1.4.3.

The ITS-1 sequence data for *Uncinaria* samples and the *cox1* sequence data for *D. nihonkaiensis* and *B. transfuga* were deposited in the National Center for Biotechnology Information GenBank database under the accession numbers LC851352–LC851355, LC851505–LC851508 and LC851509–LC851512, respectively.

### 2.7 Statistical analysis

For frequently detected species (*Uncinaria* sp., *B. transfuga*, and *D. nihonkaiensis*), a generalized liner mixed model (GLMM) was used to assess the likelihood of detection for each parasite, using samples from individually identified bears. The objective variable was parasite positivity or negativity; the explanatory variables included year, season, sex and age class. Seasons were divided into summer (late May to August) and autumn (September to November). Age classes were categorized as young (0–2 years) and subadult/adult (≥3 years). Bear individual was treated as a random effect. If several samples were obtained from the same individual during the same season in the same year, only one was used. In the case of coexisting positive and negative samples, the positive sample was selected. Thus, the model was designed to assess whether each individual was positive for parasite infection in each season (summer/autumn) of each year. All combinations of explanatory variables were tested and that with the lowest Akaike Information Criterion value was selected as the best model. Significance was evaluated at a threshold of *P* < 0.05. All statistical analyses were performed using the *glmmML* package in R v4.4.1 (R Core Team, Vienna, Austria).

## 3 Results

### 3.1 Samples

Data from 595 fecal samples were obtained and used for diet estimation (144 in 2022, 244 in 2023, and 207 in 2024). Parasitological examinations were performed from 328 fresh fecal samples and 6 fecal samples obtained from bears captured for another research purposes (Table 1; 334 samples in total, including 80 in 2022, 145 in 2023 and 109 in 2024). All fecal samples obtained in 2022 were kept frozen, as well as some of the feces (*n* = 71) obtained between August and November 2023 (for detail, see Table 1). Individual bear identification was possible from 279 (83.5%) of these feces, and 49 unique bears were identified: 33 females and 16 males during the three years of the study, including 3 young and 22 subadults/adults in 2022, 10 young and 25 subadults/adults in 2023, and 1 young and 12 subadults/adults in 2024.

**Table 1:** Numbers of fecal samples and identified bear individuals for each period.

		Estimated period of fecal excretion^a^							
		late May–early Jun.	late Jun.–early Jul.	late Jul.–early Aug.	mid–late Aug.	early–late Sep.	early–mid Oct.	late Oct.–early Nov.	Total
2022	Feces	6^d^	10^d^	7^d^	15^d^	11^d^	11^d^	20^d^	80^d^
	Bears^b^	4	5	6	11	8	8	8	25^c^
2023	Feces	16	22	22^d^	20^d^	41^e^	17	7^d^	145
	Bears^b^	9	13	11	10	16	5	2	35^c^
2024	Feces	21	22	2	15	8	19	22	109
	Bears^b^	7	6	2	4	3	5	8	13^c^

a The period of fecal excretion for each sample was determined from the date of fecal collection and the estimated number days since defecation.

b Number of unique bears identified in each period.

c Number of unique bears identified in each year.

d Fecal samples kept frozen until the analysis.

e f/4°C 22 out of 41 samples were kept frozen.

### 3.2 Parasite species

**Fig. 2:**
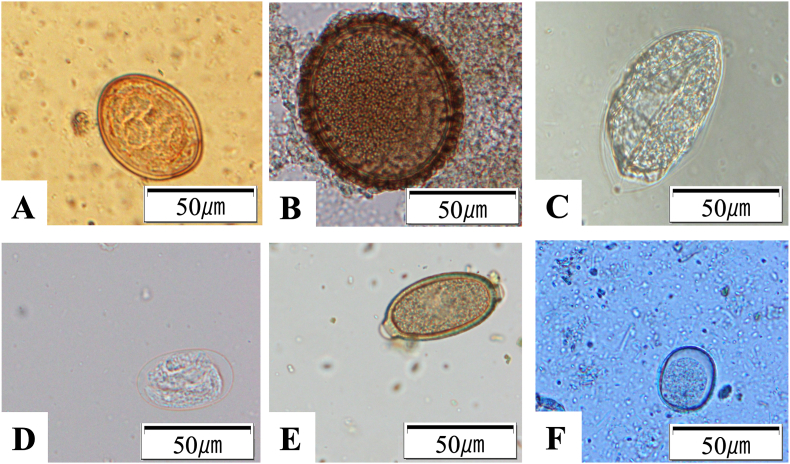
Parasites eggs and oocysts identified in brown bear feces. (A) *Dibothriocephalus nihonkaiensis* egg. (B) *Baylisascaris transfuga* eggs. (C) *Uncinaria* sp. egg. (D) Strongylida egg. (E) Capillariidae egg. (F) coccidia oocysts.

Eggs of at least one species of parasite were detected by flotation technique in 168 of 334 (50.3%) feces (Table 2, Fig. 2 and Supplementary Table S1). *Uncinaria* sp. was the most common (35.0%; 117/334), followed by *B. transfuga* (13.5%; 45/334), and *D. nihonkaiensis* (8.4%; 28/334). Eggs of Strongylida (2.7%; 9/334) and Capillariidae (0.6%; 2/334) and coccidia oocysts (0.6%; 2/334) were also found.

**Table 2:** Prevalence and mean intensity of the excretion of intestinal helminth eggs as determined in brown bear feces from eastern Hokkaido, Japan.

	2022					2023				
	Prevalence of detection		Prevalence of infection		EPG ± SD	Prevalence of detection		Prevalence of infection		EPG ± SD
*Uncinaria* sp.	10.0%	(8/80)	16.0%	(4/25)	17.1 ± 15.3	51.7%	(75/145)	62.9%	(22/35)	76.9 ± 94.8
*B. transfuga*	27.5%	(22/80)	44.0%	(11/25)	128.1 ± 189.4	11.0%	(16/145)	11.4%	(4/35)	232.8 ± 614.6
Strongylida	1.3%	(1/80)	0.0%	(0/25)	3.0 ± 0.0	3.4%	(5/145)	8.6%	(3/35)	4.2 ± 2.4
Capillariidae	0.0%	(0/80)	0.0%	(0/25)	–	0.7%	(1/145)	2.9%	(1/35)	8.0 ± 0.0
*D. nihonkaiensis*	3.8%	(3/80)	4.0%	(1/25)	290.0 ± 252.5	17.2%	(25/145)	11.4%	(4/35)	45222.0 ± 101991.3
Coccidia	0.0%	(0/80)	0.0%	(0/25)	–	1.4%	(2/145)	5.7%	(2/35)	9.0 ± 5.0

	2024					Total				
	Prevalence of detection		Prevalence of infection		EPG ± SD	Prevalence of detection		Prevalence of infection		EPG ± SD
*Uncinaria* sp.	31.2%	(34/109)	53.8%	(7/13)	28.2 ± 68.0	35.0%	(117/334)	53.1%	(26/49)	59.0 ± 88.0
*B. transfuga*	6.4%	(7/109)	38.5%	(5/13)	601.0 ± 726.7	13.5%	(45/334)	38.8%	(19/49)	238.9 ± 510.3
Strongylida	2.8%	(3/109)	15.4%	(2/13)	164.3 ± 182.2	2.7%	(9/334)	8.2%	(4/49)	57.4 ± 129.5
Capillariidae	0.9%	(1/109)	0.0%	(0/13)	10 ± 0.0	0.6%	(2/334)	2.0%	(1/49)	9.0 ± 1.0
*D. nihonkaiensis*	0.0%	(0/109)	0.0%	(0/13)	–	8.4%	(28/334)	8.2%	(4/49)	40407.9 ± 97370.0
Coccidia	0.0%	(0/109)	0.0%	(0/13)	–	0.6%	(2/334)	4.1%	(2/49)	9.0 ± 5.0

Prevalence of detection; number of feces with eggs or oocysts/total number of feces.

Prevalence of infection; number of infected bears/total number of unique bears. Bears were categorized as “infected”, if they were positive at least once in the year.

EPG; number of eggs (or oocysts for coccidia) per gram of feces. The mean EPG was calculated using only positive samples.

We obtained 411–420 bp of *cox1* sequences from four *D. nihonkaiensis* samples, 354–390 bp from four *B. transfuga* samples, and 392–432 bp of ITS-1 sequences from four *Uncinaria* samples. The sequence form one sample of *D. nihonkaiensis* (LC851508) had one single-nucleotide polymorphism, as compared to those from the other three samples (LC851505–LC851507). The sequences of the *B. transfuga* samples were identical in all four samples; the same was true for the *Uncinaria* samples. The sequences obtained from *D. nihonkaiensis* eggs showed 99.0–100% homology to the registered sequences of *D. nihonkaiensis*, those from *B. transfuga* eggs showed 96.6–98.9% homology to the registered sequences of *B. transfuga*, and those from *Uncinaria* eggs showed high homology (96.8–99.5%) to registered sequences of several species of *Uncinaria* (Supplementary Table S2). Based on the ML tree, the four *Uncinaria* samples were grouped into the same cluster as *U. rauschi* (Supplementary Fig. 1).

### 3.3 Factors determining parasite presence

**Fig. 3:**
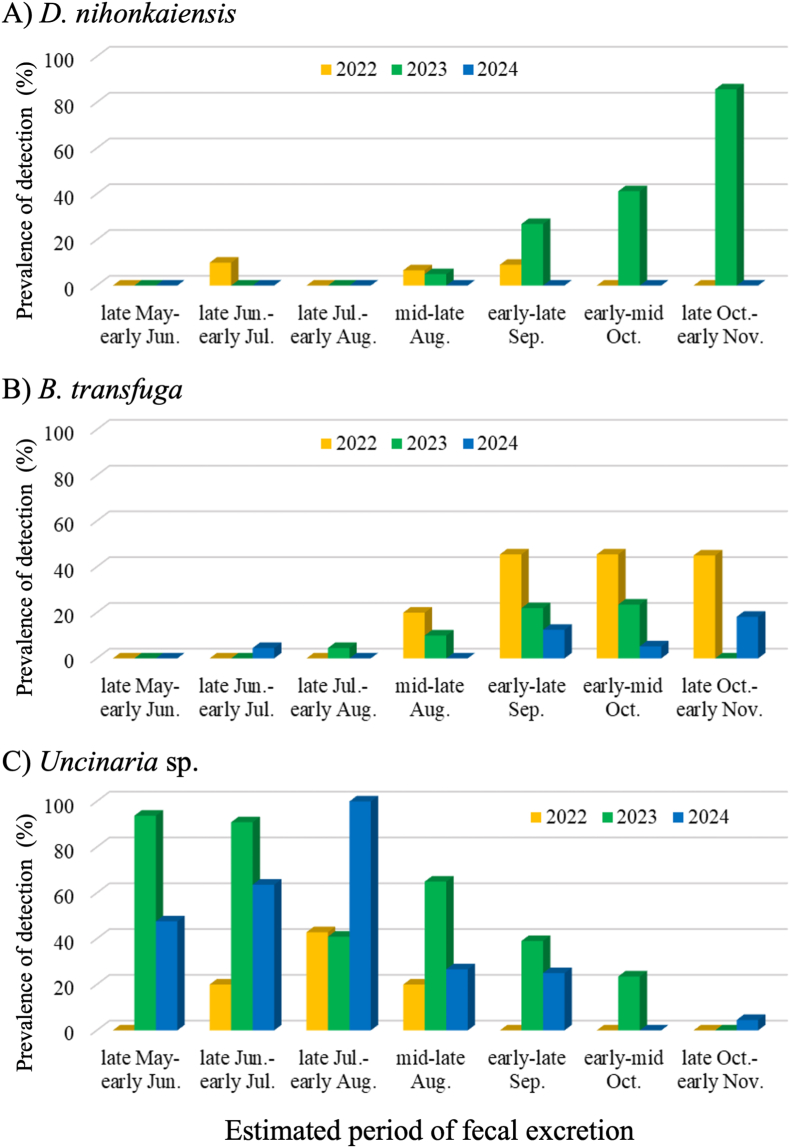
Percent prevalence of detection of parasite eggs in brown bear feces during each period between 2022 and 2024. (A) Prevalence of detection of *Dibothriocephalus nihonkaiensis*. (B) Prevalence of detection of *Baylisascaris transfuga*. (C) Prevalence of detection of *Uncinaria* sp.

Summer-autumnal and annual changes in the prevalence of detection for *D. nihonkaiensis*, *B. transfuga*, and *Uncinaria* sp. are shown in Fig. 3. For *D. nihonkaiensis*, the prevalence of detection increased from mid-late August and peaked in late October-early November in 2023, whereas there were only three positive samples in 2022 and none in 2024. Only four adult females were found to be infected during the study period. None of the tested explanatory variables were significant in predicting parasite detection in fecal samples. For *B. transfuga*, the prevalence of detection tended to increase from September onwards. The best model for the presence of *B. transfuga* included season (summer/autumn), year, and age class as explanatory variables (Table 3). The likelihood of *B. transfuga* detection was significantly higher in autumn than in summer, lower in 2023 than in other years, and tended to be higher in young than in subadult/adult bears (*P* = 0.053). In contrast to the above two parasite species, the prevalence of detection of *Uncinaria* sp. tended to be high at the beginning of the year and decreased in autumn. The best model for the presence of *Uncinaria* sp. included season (summer/autumn), year, and age class as explanatory variables (Table 3). The likelihood of *Uncinaria* sp. detection was significantly higher in summer than in autumn, lower in 2022 than in the other years, and lower in young than in subadult/adult bears.

**Table 3:** Generalized liner mixed model for the likelihood of *Baylisascaris transfuga* and *Uncinaria* sp. detection in brown bears in eastern Hokkaido, Japan.

Response variable	Explanatory variable	Estimate	SE	Z–value	Pr (>|z|)
*B. transfuga*	(Intercept)	2.036	1.140	1.787	<0.05
	Season (summer/autumn)^a^				
	summer	−2.087	0.763	−2.737	<0.01
	Year^b^				
	2023	−2.031	0.811	−2.503	<0.05
	2024	−0.454	0.817	−0.555	0.579
	Age^c^				
	subadult/adult	−2.011	1.040	−1.933	0.053
*Uncinaria* sp.	(Intercept)	−6.385	1.483	−4.307	<0.001
	Season (summer/autumn)^a^				
	summer	3.275	0.725	4.518	<0.001
	Year^b^				
	2023	3.311	0.841	3.939	<0.001
	2024	3.134	0.961	3.263	<0.01
	Age^c^				
	subadult/adult	2.035	0.890	2.286	<0.05

a Reference category for season is autumn.

b Reference category for year is 2022 (the first year of the study).

c Reference category for age class is young (0–2 years).

**Fig. 4:**
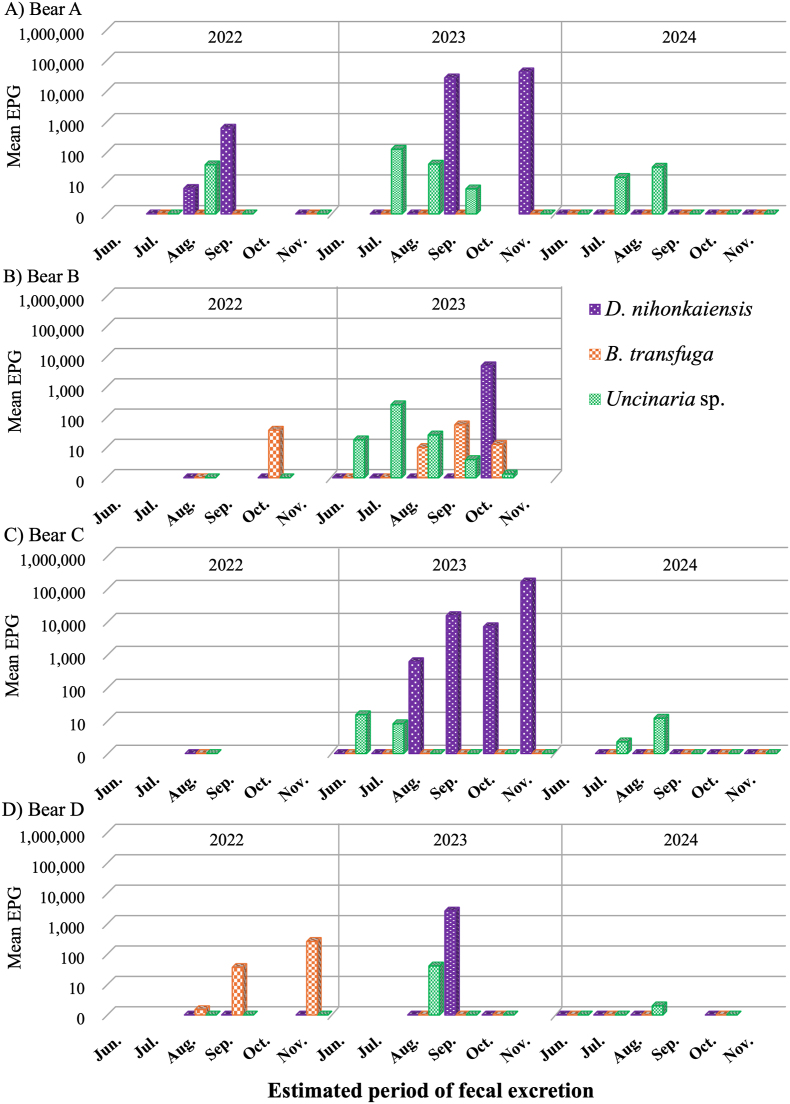
Changes in the parasite infection status in individual bears. Feces were not collected from bear B in 2024. The absence of a bar on the graph indicates that no feces were collected from that individual during that period. EPG denotes number of eggs per gram of feces.

Infection status at the individual level was determined for the four adult females (A-D) with a high frequency of fecal collection (Fig. 4). *Dibothriocephalus nihonkaiensis*, *B. transfuga*, and *Uncinaria* sp. egg shedding disappeared at the beginning or end of the year in all bears. For bear A, *D. nihonkaiensis* and *Uncinaria* sp. egg shedding disappeared before winter in 2022 and was detected again in 2023. For bear B, *B. transfuga* egg shedding was seen in 2022 but disappeared by June 2023, with eggs again detected in autumn.

### 3.4 Summer-autumnal and annual variation in diet

**Fig. 5:**
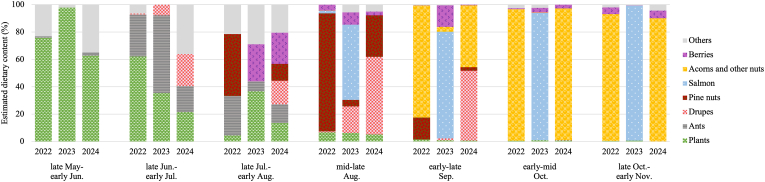
Summer-autumnal and annual variation in the dietary content of bear fecal samples, based on 595 fecal samples obtained in the Rusha area during 2022–2024.

Summer-autumnal and annual variation in the bears' diet is shown in Fig. 5 and Supplementary Table S3. Plants accounted for the highest proportion of the diet from late May to early June, after which consistent consumption was observed until August. The dietary contribution of ants was highest from late June to early July, accounting for an average of 35.5%. Drupes and berries were consumed mainly from July to September in 2023 and 2024, replaced by pine nuts during the same period in 2022. In 2023, salmon dominated the diet between mid-August and early November, particularly in October and November, when they accounted for >90% of the diet. Acorns comprised most of the diet in September or later in 2022 and 2024. Acorns and other nuts, mainly Japanese oak acorns, accounted for about 95% of the bears’ diet.

## 4 Discussion

The current study newly confirmed the presence of Strongylida, Capillariidae, and coccidia in Hokkaido brown bears. All of the detected parasites have been reported in bears in Europe and North America (Gau et al., 1999; Foster et al., 2004; Borka-Vitális et al., 2017; Bugmyrin et al., 2017). In this study, parasites eggs/oocysts were present in 50.3% of 334 samples, which is lower than the percentages for bears in Spain (79.3% of 111 feces; Remesar et al., 2024) and Slovakia (76.5% of 17 feces; Orosová et al., 2016), but equal to or higher than those reported for bears in Romania (37.9% of 211 feces; Borka-Vitális et al., 2017) and Canada (42.8% of 56 feces; Gau et al., 1999). These differences are likely due to environmental factors such as temperature, humidity, vegetation, soil type, and land use (Díaz et al., 2007; French et al., 2019), as well as host density.

*Dibothriocephalus nihonkaiensis* is an intestinal parasite that infects mammals, including bears and humans, as definitive hosts. It is transmitted by feeding on the second intermediate host, salmonids (Arizono et al., 2009). The prevalence of detection of *D. nihonkaiensis* in this study was similar to that reported in Canada (Catalano et al., 2015a). In Europe, Diphyllobothridae infections of bears are relatively rare (Orosová et al., 2016; Borka-Vitális et al., 2017; Bugmyrin et al., 2017; Remesar et al., 2024), presumably due to the low intensity of feeding on salmonids. By contrast, feeding on abundant spawning salmon is observed in coastal areas of North America (Hilderbrand et al., 1999; Mowat and Heard, 2006), while it does not occur in most brown bear habitats in of Europe (Swenson et al., 2007). In this study, the GLMM did not suggest any factors affecting the presence of *D. nihonkaiensis*, likely due to the small number of positive individuals in the dataset. However, positive cases were found only in mid-August or later, with one exception, suggesting a seasonal effect on the likelihood of detection. The prepatent period of Diphyllobothridae is considered to be 2–6 weeks (Scholz et al., 2009), indicating that this parasite is transmitted by feeding on salmon running upstream from early August (Nakamura and Komiyama, 2010). One positive case in July might be caused by preying on masu salmon (*Oncorhynchus masou masou*), returning to their home river primarily from May to June (Miyakoshi et al., 2001). Another possibility is that eggs passed through the bear intestinal tract after the bear had eaten an infected mammals (i.e., pseudo-parasitism), including cetaceans stranded on the coast (Jimbo et al., 2022).

In this study, positive cases of *D. nihonkaiensis* were biased towards 2023 but were rare in 2022 and absent in 2024. This result can be explained by differences in the amounts of foraging salmon. Salmon accounted for a high proportion of the bears’ diet from mid-August until November in 2023, whereas acorns were the main dietary content in autumn 2022 and 2024. It is unlikely that there were no opportunities for infection in 2022 and 2024, as salmon predation was observed in the field. However, poor salmon runs and an abundance of other high-calorie foods such as pine nuts in August and acorns from September to November may have reduced the chance of infection, although there are no data on the availability of each food resource.

*Baylisascaris transfuga* is one of the most common parasites in bears worldwide (Catalano et al., 2015a; Bugmyrin et al., 2017; Štrkolcová et al., 2018; Hwang et al., 2021; Haynes et al., 2023; Remesar et al., 2024). It has a direct lifecycle and is transmitted by feeding on embryonated eggs in the environment (Bauer, 2013). The prepatent period of *B. transfuga* is unknown, but is probably around 2 months, extrapolating from the closely related *B. procyonis* (Samuel et al., 2001). The likelihood of *B. transfuga* detection was significantly higher in autumn than in summer. Previous studies also reported an increasing prevalence of *B. transfuga* from spring to autumn (Molnár et al., 2020; Cano et al., 2024), which is consistent with the findings of the present study. Given the initial increase in the prevalence (August–September) of *B. transfuga* and the prepatent period of the parasite, infection is likely to be caused by food items consumed in June–July or later. Grasses and ants accounted for more than half of the dietary contents of bear fecal samples in June–July. Therefore, bears may become infected by eggs in the soil, as occurs with other *Baylisascaris* species (Bauer, 2013; Graeff-Teixeira et al., 2016), as they feed on grasses and ants. However, this explanation does not fully account for the summer-autumnal and annual differences in the likelihood of *B. transfuga* detection observed in this study, because bears also eat grasses immediately after the denning period in early spring (Sato et al., 2005a, Sato et al., 2005b), and the patterns of plants and ants used in May–August were approximately consistent each year. Studies of closely related *B. procyonis* suggested that average rainfall, temperature, and soil type influence the prevalence or presence in raccoons (Kresta et al., 2010). Thus, in this study, seasonal and annual differences in climatic conditions may have affected egg development in the environment, resulting in summer-autumnal and annual variation in the presence of eggs in bears. Another possible factor affecting the likelihood of *B. transfuga* detection is the age of the host animal. In this study, younger bears were more likely to be infected, as also reported for *B. procyonis* infections in raccoons (Jardine et al., 2014). The higher likelihood of detection in young individuals may reflect a low resistance to *Baylisascaris* species in young animals; over time, with increasing age, definitive hosts likely acquire some degree of immunity to direct infections with *Baylisascaris* species (Sapp et al., 2017).

*Uncinaria* sp. was the most common species detected in this study. Infections with *Uncinaria* species have been frequently reported in brown bears and American black bears (*Ursus americanus*) (Rausch et al., 1979; Catalano et al., 2015a; Kilinç et al., 2015), with a prevalence of 53.8% reported in brown bears in Canada (Catalano et al., 2015a). *Uncinaria* species can therefore be considered major parasites in some brown bear habitats. Two species of genus *Uncinaria*, *U. rauschi* and *U. yukonensis*, are currently known to infect bears (Seguel and Gottdenker, 2017). The phylogenetic tree analysis suggested that the parasite found in this study was closely related to *U. rauschi*. The detailed life cycle of *Uncinaria* species in bears is still unclear. The closely related *U. stenocephala* is transmitted orally by L3 larvae in the environment, mainly through food and water, and sometimes percutaneously (Štrkolcová et al., 2022). The prepatent period of *Uncinaria* species is 2–3 weeks (Marcus et al., 2014; Hauck et al., 2023). In this study, the likelihood of *Uncinaria* sp. detection was significantly higher in summer than in autumn, similar to other reports in which the prevalence of *U. stenocephala* in foxes was highest in spring (Saeed et al., 2006). For brown bears in Spain, however, the prevalence of ancylostomatids (likely belonging to *Uncinaria* sp.) was found to be highest in autumn (Cano et al., 2024), which indicates that seasonal occurrence patterns of *Uncinaria* species are inconsistent among species and/or regions. Similar to *B. transfuga*, feeding on grasses and ants is considered a potential risk of *Uncinaria* sp. infection. The shorter prepatent period of *Uncinaria* sp. than that of *B. transfuga* is a factor contributing to the former's earlier occurrence, although previous studies on related species suggest that climatic conditions, such as temperature and humidity, suitable for the growth of eggs and larvae may also play a role. In this study, subadult/adult bears were more likely to be infected with *Uncinaria* sp. than young bears, which may reflect greater opportunities for infection among subadult and adult bears, as they consume more food than smaller bears.

Several studies have shown that the prevalence of intestinal parasites in bears is lowest in spring and highest in autumn (Gau et al., 1999; Molnár et al., 2020). Bears are thought to eliminate adult helminths before hibernation, with reinfection occurring after they emerge from their dens, as bears restrict their food intake and evacuate their bowels shortly before hibernation (Orosová et al., 2016). However, one study found that the prevalence of *B. transfuga* in American black bears was highest in February, during hibernation (Reichert et al., 2024). In this study, the prevalences of *D. nihonkaiensis* and *B. transfuga* peaked during October–November but became almost zero during May–July. These patterns were clear at the individual level. Thus, in bears A and C, the EPG of *D. nihonkaiensis* were highest in November 2023, but the egg shedding disappeared in June and July of the following year. Similarly, *B*. *transfuga* was present in bear B in 2022, but was not found in June and July in 2023, although it reappeared in autumn of that year. These results are consistent with the removal of intestinal parasites before or during hibernation. This seems especially true for the Diphyllobothridae, which can survive for more than 20 years in the human intestine (Scholz et al., 2009). On the other hand, the prevalence of detection of *Uncinaria* sp. approached zero by November. Similarly at the individual level (bear A–D), egg shedding had almost disappeared between September and November. This suggests that egg shedding of *Uncinaria* sp. disappeared before the onset of hibernation, likely due to decrease in opportunities for new infections (i.e., decrease in consumption of grasses and ants since mid-August) and elimination of adult hookworm, although life span of this species is not clear.

The current study had several limitations. First, freezing the fecal samples may have affected the likelihood of parasite eggs detection. Storage at subzero temperatures was shown to reduce the likelihood of egg detection for Strongylida, *U. stenocephala*, and *Parascaris equorum* (equine ascarid nematodes), while those of canine ascarid nematodes (*Toxocara canis* and *Toxascaris leonina*) and taeniids were relatively unaffected by freezing (Schurer et al., 2014). In this study, all feces collected in 2022 were kept frozen until their examination. Damage to the eggs by prolonged freezing may have caused annual differences in the presence of *Uncinaria* sp. It is likely that *B. transfuga* was relatively unaffected by freezing, as this species had the highest prevalence in 2022. Second, the samples were biased with respect to certain sex and age classes, i.e., samples of males and young individuals were limited. Similarly, there was a bias in the sample size by year, as fecal samples taken in October 2023 or later were limited. Due to a shortage of food items in autumn 2023, many bears (>180 bears) approached human settlements in search of food and were finally killed for management purposes, which would have reduced the sample size and caused sex–age biases in this study. Finally, fecal collection was limited to the period between May and November, with no sampling conducted in spring or winter. Additionally, we only assessed the presence or absence of egg shedding in feces, without examining mature parasites in the digestive tract or feces. Although we rarely observed segments of *D. nihonkaiensis* in the feces, the frequency of their occurrence was not examined in this study. Therefore, it is likely that infections during the pre- or post-patent period were missed. Further research is needed to clarify the seasonal and annual patterns of intestinal parasite infections in bears.

## 5 Conclusions

This study showed that parasites, particularly *Uncinaria* sp., *B. transfuga*, and *D. nihonkaiensis*, are common in wild brown bears in Hokkaido, Japan. Summer-autumnal and annual variation in parasite presence can be attributed to multiple factors, including seasonal and/or annual changes in food items, hibernation, climatic conditions (temperature, etc.), and parasite life histories, particularly the prepatent period. Age was identified as an important factor affecting the presence of *B. transfuga* and *Uncinaria* sp. Some of the current findings were also supported by tracking the presence of parasites at the individual level. Notably, these investigations, which linked data to specific individuals, were made possible by the long-term monitoring conducted in the study area. As some of the parasites detected in this study cause or have the potential to cause diseases in humans and domestic animals, larger scale, sustained research on bear parasites is required.

## CRediT authorship contribution statement

**Mizuki Moriyoshi:** Writing – original draft, Methodology, Investigation, Formal analysis. **Naoki Hayashi:** Writing – review & editing, Validation, Methodology. **Nariaki Nonaka:** Writing – review & editing, Validation, Supervision, Methodology, Conceptualization. **Ryo Nakao:** Writing – review & editing, Validation, Methodology. **Masami Yamanaka:** Writing – review & editing, Investigation. **Toshio Tsubota:** Writing – review & editing, Supervision, Investigation. **Michito Shimozuru:** Writing – review & editing, Project administration, Methodology, Investigation, Funding acquisition, Formal analysis, Conceptualization.

## Ethical statement

All procedures involved in sample collection from live animals were conducted in accordance with the Guidelines for Animal Care and Use of Hokkaido University and were approved by the Animal Care and Use Committee of the Graduate School of Veterinary Medicine, Hokkaido University (Permit Number: 18–0083 and 23-0014).

## Funding

This work was supported by funding from the 10.13039/501100001691Japan Society for the Promotion of Science (JSPS) (https://www.jsps.go.jp/english/e-grants/index.html) 10.13039/501100001691KAKENHI Grant Numbers JP19K06833 & JP23K05312 to MS. This work was supported by the World-leading Innovative and Smart Education (WISE) Program (1801) from the Ministry of Education, Culture, Sports, Science, and Technology, Japan.

## Declaration of competing interest

The authors have no conflicts of interest to declare.

## References

[bib1] Arizono N., Yamada M., Nakamura-Uchiyama F., Ohnishi K. (2009). Diphyllobothriasis associated with eating raw pacific salmon. Emerg. Infect. Dis..

[bib2] Asakawa M., Mano T., Gardner S.L. (2006). First record of *Ancylostoma malayanum* (Alessandrini, 1905) from brown bears (*Ursus arctos* L.). Comp. Parasitol..

[bib3] Asakawa M. (2024). An overview on parasites from wild and captive bears in Japan. J. Hokkaido Vet. Med. Assoc..

[bib4] Bauer C. (2013). Baylisascariosis—infections of animals and humans with 'unusual' roundworms. Vet. Parasitol..

[bib5] Bojarska K., Selva N. (2012). Spatial patterns in brown bear *Ursus arctos* diet: the role of geographical and environmental factors. Mamm Rev..

[bib6] Borka-Vitális L., Domokos C., Földvári G., Majoros G. (2017). Endoparasites of brown bears in eastern transylvania, Romania. Ursus.

[bib7] Bugmyrin S.V., Tirronen K.F., Panchenko D.V., Kopatz A., Hagen S.B., Eiken H.G., Kuznetsova A.S. (2017). Helminths of brown bears (*Ursus arctos*) in the kola peninsula. Parasitol. Res..

[bib8] Cano E.V., Penteriani V., Vega I., Delgado M.d.M., González-Bernardo E., Bombieri G., Zarzo-Arias A., Fernández R.S.-A., Paz-Silva A. (2024). Influence of seasonality and biological activity on infection by helminths in Cantabrian bear. Int. J. Parasitol. Parasites Wildl..

[bib9] Catalano S., Lejeune M., Tizzani P., Verocai G., Schwantje H., Nelson C., Duignan P. (2015). Helminths of grizzly bears (*Ursus arctos*) and American black bears (*Ursus americanus*) in Alberta and British Columbia, Canada. Can. J. Zool..

[bib10] Catalano S., Lejeune M., Van Paridon B., Pagan C.A., Wasmuth J.D., Tizzani P., Duignan P.J., Nadler S.A. (2015). Morphological variability and molecular identification of *Uncinaria* spp. (Nematoda: ancylostomatidae) from grizzly and black bears: new species or phenotypic plasticity?. J. Parasitol..

[bib11] Cringoli G., Rinaldi L., Albonico M., Bergquist R., Utzinger J. (2013). Geospatial (s)tools: integration of advanced epidemiological sampling and novel diagnostics. Geospatial Health.

[bib12] Dahle B., Swenson J.E. (2003). Seasonal range size in relation to reproductive strategies in brown bears *Ursus arctos*. J. Anim. Ecol..

[bib13] Dahle B., Sørensen O.J., Wedul E.H., Swenson J.E., Sandegren F. (1998). The diet of brown bears *Ursus arctos* in central Scandinavia: effect of access to free‐ranging domestic sheep *Ovis aries*. Wildl. Biol..

[bib14] Di Salvo A.R., Chomel B.B. (2020). Zoonoses and potential zoonoses of bears. Zoonoses Public Health.

[bib15] Díaz P., Paz-Silva A., Sánchez-Andrade R., Suárez J.L., Pedreira J., Arias M., Díez-Baños P., Morrondo P. (2007). Assessment of climatic and orographic conditions on the infection by *Calicophoron daubneyi* and *Dicrocoelium dendriticum* in grazing beef cattle (NW Spain). Vet. Parasitol..

[bib16] Foster G.W., Cunningham M.W., Kinsella J.M., Forrester D.J. (2004). Parasitic helminths of black bear cubs (*Ursus americanus*) from Florida. J. Parasitol..

[bib17] French S.K., Pearl D.L., Peregrine A.S., Jardine C.M. (2019). *Baylisascaris procyonis* infection in raccoons: a review of demographic and environmental factors influencing parasite carriage. Vet. Parasitol..

[bib18] Gau R.J., Kutz S., Elkin B.T. (1999). Parasites in grizzly bears from the central Canadian Arctic. J. Wildl. Dis..

[bib19] Graeff-Teixeira C., Morassutti A.L., Kazacos K.R. (2016). Update on Baylisascariasis, a highly pathogenic zoonotic infection. Clin. Microbiol. Rev..

[bib80] Hassell J.M., Begon M., Ward M.J., Fèvre E.M. (2017). Urbanization and Disease Emergence: Dynamics at the Wildlife–Livestock–Human Interface. Trends Ecol. Evol..

[bib20] Hauck D., Raue K., Blazejak K., Hanna R.M., Elsemore D.A., Pantchev N., Strube C. (2023). Evaluation of a commercial coproantigen immunoassay for the detection of *Toxocara cati* and *Ancylostoma tubaeforme* in cats and *Uncinaria stenocephala* in dogs. Parasitol. Res..

[bib21] Haynes E., Coker S., Yabsley M.J., Niedrighaus K.D., Ramey A.M., Verocai G.G., Hilderbrand G.V., Joly K., Gustine D.D., Mangipane B., Leacock W.B., Crupi A.P., Cleveland C.A. (2023). Survey for selected parasites in Alaska brown bears (*Ursus arctos*). J. Wildl. Dis..

[bib22] Hewitt D.G., Robbins C.T. (1996). Estimating grizzly bear food habits from fecal analysis. Wildl. Soc. Bull..

[bib23] Hilderbrand G.V., Schwartz C.C., Robbins C.T., Jacoby M.E., Hanley T.A., Arthur S.M., Servheen C. (1999). The importance of meat, particularly salmon, to body size, population productivity, and conservation of North American brown bears. Can. J. Zool..

[bib24] Hokkaido Government, Japan (2022). https://www.pref.hokkaido.lg.jp/ks/skn/higuma/higuma.html.

[bib25] Hwang M.-H., Chin T.-W., Yu P.-H. (2021). Endoparasites of formosan black bears (*Ursus thibetanus formosanus*) during acorn season in yushan national park, taiwan. J. Wildl. Dis..

[bib26] Inukai T., Yamashita J. (1934). On an occurrence of *Ascaris* in the intestine of a bear, *Ursus arctos yesoensis* Lyd. Trans. Sapporo Nat. Hist. Soc..

[bib27] Irie T., Ito T., Kouguchi H., Yamano K., Uraguchi K., Yagi K., Nonaka N. (2017). Diagnosis of canine *Echinococcus multilocularis* infections by copro-DNA tests: comparison of DNA extraction techniques and evaluation of diagnostic deworming. Parasitol. Res..

[bib28] Ito S. (1980). Modified Wisconsin sugar centrifugal-flotation technique for nematode eggs in bovine feces. J. Jpn. Vet. Med. Assoc..

[bib29] Jardine C.M., Pearl D.L., Puskas K., Campbell D.G., Shirose L., Peregrine A.S. (2014). The impact of land use, season, age, and sex on the prevalence and intensity of *Baylisascaris procyonis* infections in raccoons (*Procyon lotor*) from Ontario, Canada. J. Wildl. Dis..

[bib30] Jimbo M., Ishinazaka T., Shirane Y., Umemura Y., Yamanaka M., Uno H., Sashika M., Tsubota T., Shimozuru M. (2022). Diet selection and asocial learning: natal habitat influence on lifelong foraging strategies in solitary large mammals. Ecosphere.

[bib31] Kalinowski S.T., Taper M.L., Marshall T.C. (2007). Revising how the computer program CERVUS accommodates genotyping error increases success in paternity assignment. Mol. Ecol..

[bib32] Katoh K., Standley D.M. (2013). MAFFT multiple sequence alignment software version 7: improvements in performance and usability. Mol. Biol. Evol..

[bib33] Kilinç Ö.O., Göz Y.a., Yilmaz A.B., Aslan L.m. (2015).

[bib34] Kohira M., Okada H., Nakanishi M., Yamanaka M. (2009). Modeling the effects of human-caused mortality on the brown bear population on the Shiretoko Peninsula, Hokkaido, Japan. Ursus.

[bib35] Kresta A.E., Henke S.E., Pence D.B. (2010). *Baylisascaris procyonis* in raccoons in Texas and its relationship to habitat characteristics. J. Wildl. Dis..

[bib36] Lucio-Forster A., Liotta J.L., Yaros J.P., Briggs K.R., Mohammed H.O., Bowman D.D. (2012). Morphological differentiation of eggs of *Ancylostoma caninum, Ancylostoma tubaeforme*, and *Ancylostoma braziliense* from dogs and cats in the United States. J. Parasitol..

[bib37] Marcus A.D., Higgins D.P., Gray R. (2014). Epidemiology of hookworm (*Uncinaria sanguinis*) infection in free-ranging Australian sea lion (*Neophoca cinerea*) pups. Parasitol. Res..

[bib38] Ministry of the Environment, Government of Japan (2017). https://www.rinya.maff.go.jp/hokkaido/press/siretoko/attach/pdf/220415-1.pdf.

[bib39] Miyakoshi Y., Nagata M., Sugiwaka K.-I., Kitada S. (2001). Commercial harvest of hatchery-reared masu salmon *Oncorhynchus masou* estimated by a coast-wide sampling program in Hokkaido, northern Japan, and the two-stage sampling schemes of landings. Fish. Sci..

[bib40] Molnár L., Königová A.b., Major P., Vasilková Z., Tomková M., Várady M. (2020). Seasonal pattern of prevalence and excretion of eggs of *Baylisascaris transfuga* in the brown bear (*Ursus arctos*). Animals.

[bib41] Mowat G., Heard D.C. (2006). Major components of grizzly bear diet across North America. Can. J. Zool..

[bib42] Nadler S.A., Adams B.J., Lyons E.T., DeLong R.L., Melin S.R. (2000). Molecular and morphometric evidence for separate species of *Uncinaria* (Nematoda: Ancylostomatidae) in California sea lions and northern fur seals: hypothesis testing supplants verification. J. Parasitol..

[bib81] Nadler, S.A., Hoberg, E.P., Hudspeth, D.S., Rickard, L.G., 2000b. Relationships of *Nematodirus* species and *Nematodirus battus* isolates (Nematoda: Trichostrongyloidea) based on nuclear ribosomal DNA sequences. J. Parasitol. 86, 588-601. doi: 10.1645/0022-3395(2000)086[0588:RONSAN]2.0.CO;2. 10.1645/0022-3395(2000)086[0588:RONSAN]2.0.CO;2 10864259

[bib43] Nadler S.A., Lyons E.T., Pagan C., Hyman D., Lewis E.E., Beckmen K., Bell C.M., Castinel A., DeLong R.L., Duignan P.J., Farinpour C., Huntington K.B., Kuiken T., Morgades D., Naem S., Norman R., Parker C., Ramos P., Spraker T.R., Berón-Ver B. (2013). Molecular systematics of pinniped hookworms (Nematoda: *Uncinaria*): species delimitation, host associations and host-induced morphometric variation. Int. J. Parasitol..

[bib44] Nakamura F., Komiyama E. (2010). A challenge to dam improvement for the protection of both salmon and human livelihood in Shiretoko, Japan's third Natural Heritage Site. Landsc. Ecol. Eng..

[bib45] Nakamura S., Yamazaki J., Matsumoto N., Inoue‐Murayama M., Qi H., Yamanaka M., Nakanishi M., Yanagawa Y., Sashika M., Tsubota T., Ito H., Shimozuru M. (2023). Age estimation based on blood DNA methylation levels in brown bears. Mol. Ecol. Resour.

[bib47] Ohta S., Hirata H., Maruyama Y., Ishinazaka T., Asakawa M. (2021). Prevalence of *Dibothriocephalus nihonkaiensis* of brown bears (*Ursus arctos*) in the Shiretoko peninsula, Hokkaido, Japan. Bull. Biogeogr. Soc. Jpn..

[bib49] Orosová T., Goldová M., Ciberej J., Štrkolcová G. (2016). Parasitofauna of brown bear (*Ursus arctos*) in the protected landscape area CHKO — poľana. Folia Vet..

[bib50] Persson I.L., Wikan S., Swenson J.E., Mysterud I. (2001). The diet of the brown bear *Ursus arctos* in the Pasvik Valley, northeastern Norway. Wildl. Biol..

[bib51] Rausch R.L., Krechmar A.V., Rausch V.R. (1979). New records of helminths from the brown bear, *Ursus arctos* L., in the soviet far east. Can. J. Zool..

[bib52] Reichert N.S., Mathieu D., Katz C.J., Hatch K.A. (2024). Exposure of American black bears (*Ursus americanus*) to ticks, tick-borne diseases, and intestinal parasites in Wisconsin. Diversity.

[bib53] Remesar S., Busto C., Díaz P., Rivas Ó., López-Bao J.V., Ballesteros F., García-Dios D. (2024). Presence of gastrointestinal and bronchopulmonary parasites in Cantabrian brown bears. Eur. J. Wildl. Res..

[bib54] Ronquist F., Teslenko M., van der Mark P., Ayres D.L., Darling A., Höhna S., Larget B., Liu L., Suchard M.A., Huelsenbeck J.P. (2012). MrBayes 3.2: efficient Bayesian phylogenetic inference and model choice across a large model space. Syst. Biol..

[bib55] Saeed I., Maddox-Hyttel C., Monrad J., Kapel C.M. (2006). Helminths of red foxes (*Vulpes vulpes*) in Denmark. Vet. Parasitol..

[bib56] Samuel W.M., Pybus M.J., Kocan A.A. (2001).

[bib57] Sapp S.G.H., Gupta P., Martin M.K., Murray M.H., Niedringhaus K.D., Pfaff M.A., Yabsley M.J. (2017). Beyond the raccoon roundworm: the natural history of non-raccoon *Baylisascaris* species in the New World. Int. J. Parasitol. Parasites Wildl..

[bib58] Sasaki M., Ishinazaka T., Nose T., Asakawa M., Nakao M. (2019). *Dibothriocephalus nihonkaiensis* infection with *Ursus arctos* in shari, Hokkaido, Japan. J. Jap. Zoo wildl. Médica Sur.

[bib59] Sato H., Une Y., Kawakami S., Saito E., Kamiya H., Akao N., Furuoka H. (2005). Fatal *Baylisascaris* larva migrans in a colony of Japanese macaques kept by a safari-style zoo in Japan. J. Parasitol..

[bib60] Sato Y. (2017). The future of urban brown bear management in Sapporo, Hokkaido, Japan: a review. Mamm. Stud..

[bib61] Sato Y., Kobayashi Y., Urata T., Takatsuki S. (2008). Home range and habitat use of female brown bear (*Ursus arctos*) in Urahoro, eastern Hokkaido, Japan. Mamm. Study.

[bib62] Sato Y., Mano T., Takatsuki S. (2005). Stomach contents of brown bears *Ursus arctos* in Hokkaido, Japan. Wildl. Biol..

[bib63] Scholz T.S., Garcia H.H., Kuchta R., Wicht B. (2009). Update on the human broad tapeworm (Genus *Diphyllobothrium*), including clinical relevance. Clin. Microbiol. Rev..

[bib64] Schurer J., Davenport L., Wagner B., Jenkins E. (2014). Effects of sub-zero storage temperatures on endoparasites in canine and equine feces. Vet. Parasitol..

[bib65] Seguel M., Gottdenker N. (2017). The diversity and impact of hookworm infections in wildlife. Int. J. Parasitol. Parasites Wildl..

[bib66] Shimozuru M., Jimbo M., Adachi K., Kawamura K., Shirane Y., Umemura Y., Ishinazaka T., Nakanishi M., Kiyonari M., Yamanaka M., Amagai Y., Ijuin A., Sakiyama T., Kasai S., Nose T., Shirayanagi M., Tsuruga H., Mano T., Tsubota T., Fukasawa K., Uno H. (2022). Estimation of breeding population size using DNA‐based pedigree reconstruction in brown bears. Ecol. Evol..

[bib67] Shimozuru M., Shirane Y., Tsuruga H., Yamanaka M., Nakanishi M., Ishinazaka T., Kasai S., Nose T., Masuda Y., Fujimoto Y., Mano T., Tsubota T. (2019). Incidence of multiple paternity and inbreeding in high-density brown bear populations on the Shiretoko Peninsula, Hokkaido, Japan. J. Hered..

[bib68] Shimozuru M., Yamanaka M., Nakanishi M., Moriwaki J., Mori F., Tsujino M., Shirane Y., Ishinazaka T., Kasai S., Nose T., Masuda Y., Tsubota T. (2017). Reproductive parameters and cub survival of brown bears in the Rusha area of the Shiretoko Peninsula, Hokkaido, Japan. PLoS One.

[bib69] Shirane Y., Jimbo M., Yamanaka M., Nakanishi M., Mori F., Ishinazaka T., Sashika M., Tsubota T., Shimozuru M. (2021). Dining from the coast to the summit: salmon and pine nuts determine the summer body condition of female brown bears on the Shiretoko Peninsula. Ecol. Evol..

[bib70] Stenset N.E., Lutnæs P.N., Bjarnadóttir V., Dahle B.R., Fossum K.H.I., Jigsved P., Johansen T., Neumann W., Opseth O., Rønning O., Steyaert S.M.J.G., Zedrosser A., Brunberg S., Swenson J.E. (2016). Seasonal and annual variation in the diet of brown bears *Ursus arctos* in the boreal forest of southcentral Sweden. Wildl. Biol..

[bib71] Stien A., Irvine R.J., Ropstad E., Halvorsen O., Langvatn R., Albon S.D. (2002). The impact of gastrointestinal nematodes on wild reindeer: experimental and cross‐sectional studies. J. Anim. Ecol..

[bib72] Støen O.-G., Bellemain E., Sæbø S., Swenson J.E. (2005). Kin-related spatial structure in brown bears *Ursus arctos*. Behav. Ecol. Sociobiol..

[bib73] Štrkolcová G., Goldová M., Šnábel V., Špakulová M., Orosová T., Halán M., Mojžišová J. (2018). A frequent roundworm *Baylisascaris transfuga* in overpopulated brown bears (*Ursus arctos*) in Slovakia: a problem worthy of attention. Acta Parasitol..

[bib74] Štrkolcová G., Mravcová K., Mucha R., Mulinge E., Schreiberová A. (2022). Occurrence of hookworm and the first molecular and morphometric identification of *Uncinaria stenocephala* in dogs in Central Europe. Acta Parasitol..

[bib75] Swenson J.E., Adamič M., Huber D., Stokke S. (2007). Brown bear body mass and growth in northern and southern Europe. Oecologia.

[bib76] Tanabe A.S. (2008). MrBayes5D. https://www.fifthdimension.jp/.

[bib77] Thompson R.C.A., Jymbery A.J., Smith A. (2010). Parasites, emerging disease and wildlife conservation. Int. J. Parasitol..

[bib78] Wicht B., de Marval F., Peduzzi R. (2007). *Diphyllobothrium nihonkaiense* (Yamane et al., 1986) in Switzerland: first molecular evidence and case reports. Parasitol. Int..

[bib79] Yamamoto K., Tsubota T., Komatsu T., Katayama A., Murase T., Kita I., Kudo T. (2002). Sex identification of Japanese black bear, *Ursus thibetanus japonicus*, by PCR based on amelogenin gene. J. Vet. Med. Sci..

