# Supplementary material for: Patterns of intestinal parasite prevalence in brown bears (Ursus arctos) revealed by a 3-year survey on the Shiretoko peninsula, Hokkaido, Japan
Source: Int J Parasitol Parasites Wildl. 2025 Feb 19;26:101048. doi: 10.1016/j.ijppaw.2025.101048 (PMC11910688; doi:10.1016/j.ijppaw.2025.101048)
Supplement: Multimedia component 1 [file mmc1.pdf]

Supplemental Information for:

Patterns of intestinal parasite prevalence in brown bears (*Ursus arctos*) revealed by a 3-year survey on the Shiretoko Peninsula, Hokkaido, Japan

Mizuki Moriyoshi[1], Naoki Hayashi[2, 3], Nariaki Nonaka[2, 3, 4], Ryo Nakao[2, 3, 4], Masami Yamanaka[5], Toshio Tsubota[1], Michito Shimozuru[1, 4]

Table of Contents:

Supplementary Table S1: Page 2

Supplementary Table S2: Page 3

Supplementary Table S3: Page 4

Supplementary Figure S1: Page 5

Supplementary Table S1. Prevalence of single and mixed infections of *Dibothriocephalus nihonkaiensis*, *Baylisascaris transfuga*, and *Uncinaria* sp.

		late May-early Jun.		late Jun.-early Jul.		late Jul.-early Aug.		mid-late Aug.		early-late Sep.		early-mid Oct.		late Oct.-early Nov.	
		PD#	Pos/N*	PD#	Pos/N*	PD#	Pos/N*	PD#	Pos/N*	PD#	Pos/N*	PD#	Pos/N*	PD#	Pos/N*
D. nihonkaiensis	2022	0.0%	0/6	0.0%	0/10	0.0%	0/7	0.0%	0/15	9.1%	1/11	0.0%	0/11	0.0%	0/20
	2023	0.0%	0/17	0.0%	0/22	0.0%	0/23	5.0%	1/20	22.0%	9/41	17.6%	3/17	85.7%	6/7
	2024	0.0%	0/21	0.0%	0/22	0.0%	0/2	0.0%	0/15	0.0%	0/8	0.0%	0/19	0.0%	0/22
B. transfuga	2022	0.0%	0/6	0.0%	0/10	0.0%	0/7	13.3%	2/15	45.5%	5/11	45.5%	5/11	45.0%	9/20
	2023	0.0%	0/17	0.0%	0/22	0.0%	0/23	5.0%	1/20	4.9%	2/41	0.0%	0/17	0.0%	0/7
	2024	0.0%	0/21	0.0%	0/22	0.0%	0/2	0.0%	0/15	12.5%	1/8	5.3%	1/19	18.2%	4/22
Uncinaria sp.	2022	0.0%	0/6	10.0%	1/10	42.9%	3/7	6.7%	1/15	0.0%	0/11	0.0%	0/11	0.0%	0/20
	2023	88.2%	15/17	90.9%	20/22	34.8%	8/23	60.0%	12/20	17.1%	7/41	5.9%	1/17	0.0%	0/7
	2024	47.6%	10/21	63.6%	14/22	100%	2/2	26.7%	4/15	25.0%	2/8	0.0%	0/19	4.5%	1/22
D. nihonkaiensis + *B. transfuga*	2022	0.0%	0/6	0.0%	0/10	0.0%	0/7	0.0%	0/15	0.0%	0/11	0.0%	0/11	0.0%	0/20
	2023	0.0%	0/17	0.0%	0/22	0.0%	0/23	0.0%	0/20	0.0%	0/41	5.9%	1/17	0.0%	0/7
	2024	0.0%	0/21	0.0%	0/22	0.0%	0/2	0.0%	0/15	0.0%	0/8	0.0%	0/19	0.0%	0/22
D. nihonkaiensis + *Uncinaria* sp.	2022	0.0%	0/6	10.0%	1/10	0.0%	0/7	6.7%	1/15	0.0%	0/11	0.0%	0/11	0.0%	0/20
	2023	0.0%	0/17	0.0%	0/22	0.0%	0/23	0.0%	0/20	4.9%	2/41	0.0%	0/17	0.0%	0/7
	2024	0.0%	0/21	0.0%	0/22	0.0%	0/2	0.0%	0/15	0.0%	0/8	0.0%	0/19	0.0%	0/22
B. transfuga + *Uncinaria* sp.	2022	0.0%	0/6	0.0%	0/10	0.0%	0/7	6.7%	1/15	0.0%	0/11	0.0%	0/11	0.0%	0/20
	2023	0.0%	0/17	0.0%	0/22	4.3%	1/23	5.0%	1/20	17.1%	7/41	0.0%	0/17	0.0%	0/7
	2024	0.0%	0/21	4.5%	1/22	0.0%	0/2	0.0%	0/15	0.0%	0/8	0.0%	0/19	0.0%	0/22
D. nihonkaiensis + *B. transfuga* + *Uncinaria* sp.	2022	0.0%	0/6	0.0%	0/10	0.0%	0/7	0.0%	0/15	0.0%	0/11	0.0%	0/11	0.0%	0/20
	2023	0.0%	0/17	0.0%	0/22	0.0%	0/23	0.0%	0/20	0.0%	0/41	17.6%	3/17	0.0%	0/7
	2024	0.0%	0/21	0.0%	0/22	0.0%	0/2	0.0%	0/15	0.0%	0/8	0.0%	0/19	0.0%	0/22

#PD denotes percent prevalence of detection of parasite eggs in brown bear feces.

*Pos/N denotes number of positive samples per number of feces obtained in each period.

Supplementary Table S2. Homology between DNA sequences of the samples and registered sequences.

	Sample ID	Accession number	Length	*D. nihonkaiensis* (*cox1*)	*B. transfuga* (*cox1*)	*U. rauschi* (ITS-1)	*U. yukonensis* (ITS-1)	*U. stenocephala* (ITS-1)
Diphyllobothriidae	220928HUS4	LC851505	420 bp	99.3–100%				
	230913HUS4	LC851506	420 bp	99.3–100%				
	230929HUS2	LC851507	411 bp	99.3–100%				
	220705RU-Bd4	LC851508	411 bp	99.0–100%				
Roundworm	230930HUS11	LC851509	376 bp		97.1–98.9%			
	221107HUS1	LC851510	354 bp		96.6–98.9%			
	221017RU-Bd5	LC851511	388 bp		97.8–98.7%			
	240703HS-Bd1	LC851512	390 bp		96.7–98.7%			
Hookworm	230607HUS1	LC851352	432 bp			99.5%	98.8%	96.8–99.1%
	230704HUS3	LC851353	392 bp			99.4–99.5%	98.6–98.7%	97.1–99.2%
	230704HUS7	LC851354	432 bp			99.5%	98.8%	96.8–99.1%
	230912HS-Bd1	LC851355	432 bp			99.5%	98.8%	96.8–99.1%

cox1: cytochrome c oxidase subunit 1; ITS-1: internal transcribed spacer 1

Supplementary Table S3. Percent volume and frequency of occurrence of each food item detected among 595 fecal samples obtained during 2022–2024.

		late May-early Jun.		late Jun.-early Jul.		late Jul.-early Aug.		mid-late Aug.		early-late Sep.		early-mid Oct.		late Oct.-early Nov.	
		Volume	Frequency	Volume	Frequency	Volume	Frequency	Volume	Frequency	Volume	Frequency	Volume	Frequency	Volume	Frequency
Plants	2022	75.7%	6/6	62.2%	20/21	4.4%	6/12	6.6%	6/20	1.5%	7/31	0.5%	2/26	0.3%	1/29
	2023	97.9%	18/18	35.4%	3/24	36.7%	25/31	6.2%	22/35	0.9%	22/90	0.9%	17/36	0.6%	6/10
	2024	62.7%	29/29	21.5%	35/45	13.5%	10/15	5.1%	8/28	0.6%	3/36	0.7%	3/30	0.0%	0/27
Ants	2022	1.1%	1/6	30.7%	6/21	28.8%	5/12	0.0%	0/20	0.0%	0/31	0.0%	0/26	0.0%	0/29
	2023	0.0%	0/18	56.9%	15/24	7.4%	2/31	0.0%	2/35	0.0%	1/90	0.0%	0/36	0.0%	0/10
	2024	2.4%	1/29	19.1%	11/45	13.7%	2/15	0.0%	1/28	0.0%	0/36	0.0%	0/30	0.0%	0/27
Drupes	2022	0.0%	0/6	0.7%	1/21	0.0%	0/12	0.7%	1/20	0.0%	0/31	0.0%	0/26	0.0%	0/29
	2023	0.0%	0/18	7.7%	1/24	0.0%	0/31	19.5%	12/35	1.3%	3/90	0.0%	0/36	0.0%	0/10
	2024	0.0%	0/29	23.3%	7/45	17.2%	1/15	56.9%	15/28	51.2%	19/36	0.3%	2/30	0.0%	0/27
Pine nuts	2022	0.0%	0/6	0.0%	0/21	45.2%	5/12	86.3%	14/20	16.0%	6/31	0.0%	0/26	0.0%	0/29
	2023	0.0%	0/18	0.0%	0/24	0.0%	0/31	4.6%	2/35	0.0%	0/90	0.0%	0/36	0.0%	0/10
	2024	0.0%	0/29	0.0%	0/45	12.3%	2/15	30.4%	10/28	2.6%	1/36	0.0%	0/30	0.0%	0/27
Salmon	2022	0.0%	0/6	0.0%	0/21	0.0%	0/12	1.9%	1/20	0.0%	0/31	0.0%	0/26	0.0%	0/29
	2023	0.0%	0/18	0.0%	0/24	0.0%	0/31	55.1%	1/35	78.0%	13/90	93.1%	10/36	98.7%	7/10
	2024	0.0%	0/29	0.0%	0/45	0.0%	0/15	0.0%	0/28	0.0%	0/36	0.0%	0/30	0.0%	0/27
Acorns and other nuts	2022	0.0%	0/6	0.0%	0/21	0.0%	0/12	0.1%	1/20	81.9%	23/31	96.1%	26/26	92.8%	27/29
	2023	0.0%	0/18	0.0%	0/24	0.0%	0/31	0.0%	0/35	3.5%	14/90	0.3%	2/36	0.0%	0/10
	2024	0.0%	0/29	0.0%	0/45	0.0%	0/15	0.0%	0/28	45.2%	20/36	96.2%	29/30	90.1%	24/27
Berries	2022	0.0%	0/6	0.0%	0/21	0.1%	1/12	4.3%	2/20	0.1%	3/31	0.8%	6/26	4.8%	7/29
	2023	0.0%	0/18	0.0%	0/24	27.0%	12/31	8.9%	14/35	15.7%	78/90	3.4%	20/36	0.0%	1/10
	2024	0.0%	0/29	0.0%	0/45	22.7%	5/15	2.6%	5/28	0.5%	6/36	2.8%	7/30	5.6%	9/27
Others	2022	23.2%	3/6	6.4%	3/21	21.5%	2/12	0.1%	2/20	0.5%	1/31	2.6%	1/26	2.0%	0/29
	2023	2.1%	1/18	0.0%	0/24	28.9%	8/31	5.7%	7/35	0.5%	3/90	2.4%	5/36	0.6%	2/10
	2024	34.9%	5/29	36.1%	17/45	20.5%	4/15	5.1%	2/28	0.0%	0/36	0.0%	0/30	4.3%	1/27

Volume; percent fecal volume for each food item corrected by applying correction factors.

Frequency; number of feces containing each food / number of fecal data

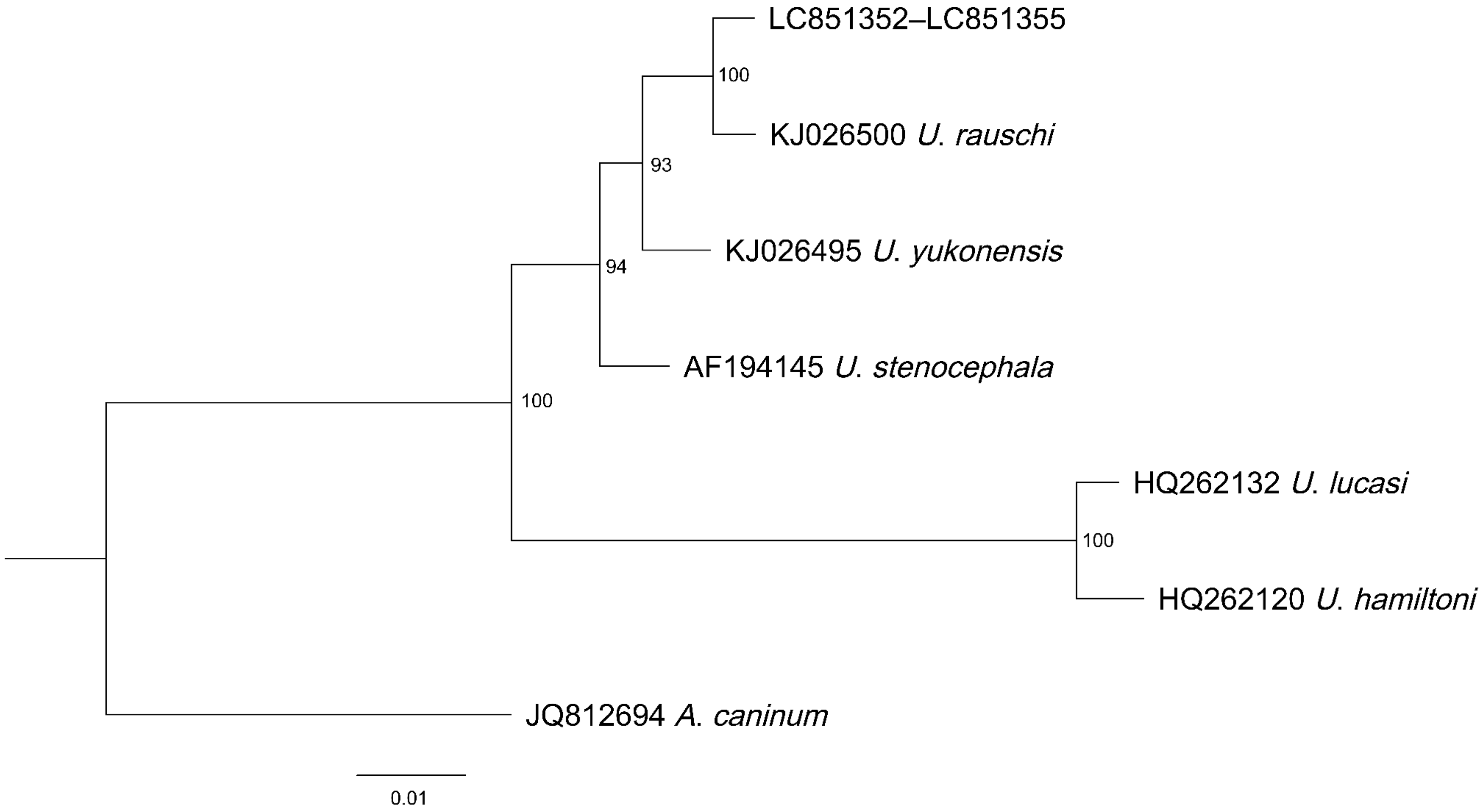

Supplementary Fig. S1. A Bayesian phylogenetic tree inferred from internal transcribed spacer 1 (ITS-1) ribosomal DNA (rDNA) datasets of *Uncinaria* sp. (including 4 samples; LC831352– LC851355) and *Ancylostoma caninum* (outgroup). Values on nodes represent the posterior probabilities.
